# What we don't need to prove but need to do in multidisciplinary treatment and care in Huntington's disease: a position paper

**DOI:** 10.1186/s13023-023-02622-8

**Published:** 2023-01-30

**Authors:** Alzbeta Mühlbӓck, Marleen van Walsem, Martha Nance, Astri Arnesen, Kirsty Page, Alexandra Fisher, Manon van Kampen, Angela Nuzzi, Roy Limpert, Hanne Ludt Fossmo, Travis Cruickshank, Ruth Veenhuizen, the Multidisciplinary Care and Treatment Working Group of the European Huntington’s Disease Network

**Affiliations:** 1 Department of Neurology, University Ulm, Ulm, Germany; 2 Department of Neuropsychiatry, Huntington Center South, Kbo-Isar-Amper-Klinikum Taufkirchen, Taufkirchen, Germany; 3 Department of Neurology and Center of Clinical Neuroscience, 1st Faculty of Medicine, Charles University and General University Hospital, Prague, Czech Republic; 4 Department of Neurorehabilitation, Oslo University Hospital, Oslo, Norway; 5 Department of Neurology, Oslo University, Oslo, Norway; 6 Struthers Parkinson’s Center, Golden Valley, Minneapolis, MN USA; 7 Hennepin County Medical Center, Minneapolis, MN USA; 8 European Huntington Association (EHA), Moerbeke Waas, Belgium; 9 Inspire Neurocare, Worcester, UK; 10 West Midlands Huntington’s Disease Team, Neuropsychiatry, The Barberry, Birmingham, UK; 11 Huntington Expert Centre Atlant, Apeldoorn, The Netherlands; 12 Rehabilitation Unit DAS, ASL Bari, Bari, Italy; 13 Unit for Congenital and Hereditary Neuromuscular Disorders (EMAN), Department of Neurology, Oslo University Hospital, Oslo, Norway; 14 Vikersund Rehabilitation Centre, Vikersund, Norway; 15 Centre for Precision Health, Edith Cowan University, Perth, Australia; 16 Department of Medicine for Older People, Amsterdam UMC, Location Vrije Universiteit Amsterdam, Amsterdam, The Netherlands; 17 Amsterdam Public Health, Aging and Later Life, Amsterdam, The Netherlands

**Keywords:** Huntington’s disease, Multidisciplinary treatment, Care, Managed care network, Case manager, Guidelines, Position paper, Interdisciplinary team

## Abstract

**Background:**

Huntington’s disease is a complex neurodegenerative hereditary disease with symptoms in all domains of a person’s functioning. It begins after a healthy start in life and leads through the relentless progression over many years to complete care dependency and finally death. To date, the disease is incurable. The long progressive complex nature of the disease demands multiple disciplines for treatment and care of patient and family. These health care providers need inter- and multidisciplinary collaboration to persevere and be efficacious in this devastating disease trajectory.

**Discussion:**

The position paper outlines current knowledge and experience alongside the experience and consensus of a recognised group of HD multidisciplinary experts. Additionally the patient’s voice is clear and calls for health care providers with a holistic view on patient and family. Building long-term trust is a cornerstone of the network around the patient. This paper describes a managed care network comprising all the needed professionals and services. In the health care system, the role of a central coordinator or case manager is of key importance but lacks an appropriate guideline. Other disciplines currently without guidelines are general practitioners, nurses, psychologists, and social workers. Guidelines for neurologists, psychiatrists, geneticists, occupational therapists, speech and language therapists, physiotherapists, dieticians, and dentists are being discussed. Apart from all these profession-specific guidelines, distinctive inter- and multidisciplinary collaboration requirements must be met.

**Conclusions and recommendations:**

The complex nature of Huntington's disease demands multidisciplinary treatment and care endorsed by international regulations and the lay association. Available guidelines as reviewed in this paper should be used, made available by a central body, and updated every 3–5 years. Time needs to be invested in developing missing guidelines but the lack of this ‘proof’ should not prevent the ‘doing’ of good care.

## Introduction

**Fig. 1:**
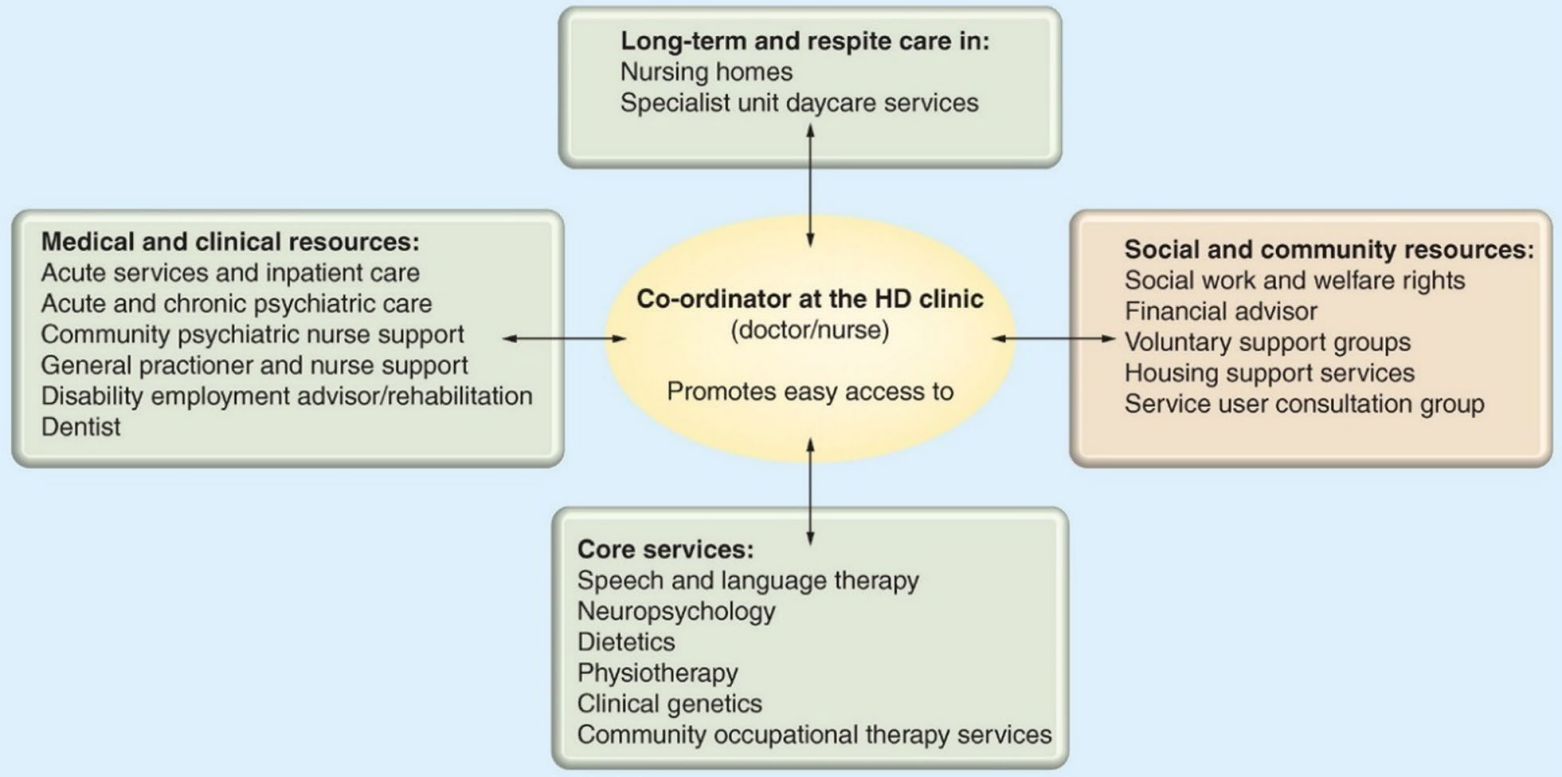
Concept of the managed care network in HD in which the coordinator is the central person for the family and the whole network

The Multidisciplinary Treatment and Care Working Group (MTC WG) of the European Huntington's Disease Network (EHDN) aims to improve treatment and care for patients and families affected by Huntington's Disease (HD). The full trajectory from being born in an HD family to the advanced stages of the disease and finally death needs to be provided with expert guidance, counselling, treatment, and care. As HD is a family disease, we include family members when we speak about people living with HD. In subsequence to the Standards of Care described by Simpson and Rae [1] (members of the 'Standards of Care' working group of EHDN), this position paper is based on the longstanding expertise of the Multidisciplinary Treatment and Care Working Group of EHDN, which represents several professional groups involved in the care of patients and their families in clinical practice. The position paper draws on the concept of a managed care network [2], as shown in Fig. 1. The aim is to outline where we currently stand and what we already know about day-to-day multidisciplinary treatment and care for HD families and to provide directions for the future. A consensus statement was formulated after reviewing available literature and available guidelines by the MTC WG of the EHDN. This position statement acknowledges what is already available and therefore needs no further proof before implementation. This paper is written for health care providers within and outside the HD communities/centres of expertise.

### Huntington’s disease

Huntington's disease (HD) is caused by an elongated CAG triplet repeat expansion in exon 1 of the Huntingtin gene in chromosome 4 [3]. Despite the tiny distinct location, this dominantly inherited disease shows a complex pattern of symptoms, course and age of onset. HD patients suffer from motor, cognitive, psychiatric and general symptoms, with disease symptoms typically beginning in the 3rd-4th decades of life, but sometimes at a much younger or older age, progressing over 10–20 years to death [4]. The majority of patients have symptoms in all these domains over the disease course, in variable combinations. The first manifestations typically occur with non-specific psychiatric and cognitive changes [5]. However, these symptoms can only facilitate the diagnosis of HD if there is a positive family history. Otherwise, the diagnosis is confirmed in most cases by the appearance of motor symptoms that comprise involuntary choreic movements, dystonia, incoordination and an eventual moderate to a marked slowing of intended movements [4]. In combination, these motor impairments have debilitating consequences for gait, balance, posture, manual dexterity, swallowing and speech [6]. The clinical practice shows that more patients than estimated, with up to 98%, experience psychiatric complaints to some extent during the disease, either transiently or continuously [7, 8]. The different stages present different challenges. Symptoms include depressive moods, anxiety, reduced activity, poor self-care and lack of initiative, which can persist over time and develop into severe apathy affecting daily functioning and quality of life. Besides apathy as the most frequent symptom, irritability, poor temper control, impaired judgment, behavioural inflexibility, aggression, suicidal ideations, delusions, psychosis, and obsessive–compulsive behaviour (OCD) may be prevalent in all stages of HD [8, 9], which can be hard to treat and detrimental to social functioning and wellbeing.

However, cognitive disorders that are not notable at first sight are critical for effective environmental functioning [10]. The cognitive decline includes problems in verbal learning, attentional difficulties, and executive and social cognitive dysfunction, which is frequently accompanied by diminished awareness of symptoms by the patient, which creates tension and conflict within the family and interferes with medical care. Finally, more general features such as weight loss, sleep and circadian rhythm disturbance, and autonomic dysfunction add to the complexity of HD.

We believe that optimal care for this complex genetic disease cannot be provided by a single health care provider (HCP) but requires a comprehensive and multidisciplinary approach, adjusted to the patients' and proxies’ individual needs and depending on the stage of the disease.

### Patient's voice

Studies show that HD patients and families have great unmet needs [11, 12]. From the patient community, it is regarded as a paradox that all the knowledge and good practices in HD that have been developed over the past 20–30 years are being used by only a small proportion of HCPs. This paper points to the value of the existing knowledge and encourages HCPs to use this. It would be of great importance if the patient affected by Huntington's disease were to encounter an HCP who would be willing to use the knowledge already available. As explained in the following sections, there are some principles that HCPs should keep in mind in order to provide quality services to families and patients living with HD.

### A holistic patient and family-centred approach is needed

The fact that HD is inheritable, progressive and presents with a wide range of symptoms makes a holistic approach imperative. There is never just one symptom or problem that needs to be addressed. A holistic approach requires the HCP to look beyond their own expertise. Moreover, good collaboration and coordination between multidisciplinary health care services are compulsory. We would argue that individuals living with HD and their families have their own separate needs for health care and support, as well as family dynamics. Therefore, we would propose that patient-centred care needs to be defined as a family-centred approach.

### Long-term trust-building is pivotal

The fact that HD is a complex neurodegenerative disease with cognitive decline and psychiatric problems complicated by stigma, shame, and fear of what will come creates a paradoxical situation for the health care system. Patients and family members have a lot of need for health care and support, but often the patients tend to underestimate their symptoms and consequences. Furthermore, family members struggle to make their needs explicit. HD families need to meet patient-oriented, empathetic, and proactive HCPs. Long-term trust building includes getting to know the patient and family and learning about their needs and unsolved issues [13]. Acknowledging and emphasising the patient and family members' experience with the disease, i.e. what is causing challenges and problems for the individual and the family is pivotal. Subsequently, joint decision-making on treatment agreements can help clarify things for the patient and increase adherence to a plan.

### Regulatory standards

The core principles listed in the former section can be seen in the light of the World Health Organisation (WHO) definition of good quality care [14], as these principles emphasise the need to provide safe, evidence-based healthcare services which are people-centred, timely and integrated throughout the disease course. Evidence-based medicine (EBM) is the conscientious, explicit, judicious and reasonable use of current best evidence in making decisions about the care of individual patients [15]. EBM integrates clinical experience and patient values with the best available research information.

This definition resounds in the European Reference Network for Rare Neurological Diseases (ERN-RND), for which the European Huntington Association (EHA) has developed a Patient Journey for HD in close collaboration with patient representatives. This endorsed document underlines the same principles as we do in this paper [16, 17].

### Guidelines

The following section details the latest guidelines regarding strategies to address clinical aspects of HD, usually delivered by members of a specialist multidisciplinary team (MDT). Guidelines may include assessments and interventions. This article defines guidelines as practice documents that summarise the best available evidence in combination with expert consensus. Furthermore, the guidelines referred to in this article are those recognised, commissioned through, and used by HD-specific clinical and patient organisations. The composition of professions in MDTs for HD varies considerably worldwide, as do services available to people with HD and their families. We address different topics from the perspective of HD management by referring to guidelines that, no matter how the local structure of care is provided, those unfamiliar with the condition may use the information in a practical way. We also highlight that because of the latter, in combination with the complex nature of the condition, HD specialists often practise an extended scope drawing on interdisciplinary skills that may fall outside of how their typical remit may be viewed. Whilst not every profession is represented in the task force which produced this section, their professional networks within the HD community were used to gather the information, and this knowledge has guided the flow of this section.

## Clinical management

### Neurology

The reinvigoration of this multi and interdisciplinary model is illustrated through the latest synthesis of the scientific evidence for the treatment of HD fronted by the EHDN Task Force and written by Bachoud-Levi et al. [18]. As well as being a current consensus on practice, the international accord in this document is geared towards ascertaining the quality of the published literature on tested treatment and guiding readers towards this and the gaps in knowledge. These are referred to as neurological guidelines for the purpose of this chapter. In contrast, it encompasses the breadth of the complex range of symptoms seen in the condition, from bruxism to hypersalivation to sexual behaviour.

### Neuropsychiatry

Neuropsychiatrists recognise that the mental disorder experienced by people with HD is a combination of both brain dysfunction and the psychological aspects of the illness; their treatment modality lends itself to this fact. Neuropsychiatric symptoms will often have the greatest detriment to the quality of life of the person and their family [19]. However, Van Duijn et al.’s [20] analysis clustered around apathy, depression, obsessive–compulsive behaviours, irritability and sleep disorder; Eddy et al. stressed the inclusion of insight, anxiety, suicidality, impulsivity, disinhibition, psychosis and social cognition [19]. Management of neuropsychiatric symptoms in HD follows the pharmacological and non-pharmacological strategies recommended for other populations [19]. Specialist knowledge forms the basis of the consensus guidelines [21] rather than an experimental appraisal. This expertise considers the co-occurrence of these manifestations with other disease symptoms. Quigley [22] provides additional considerations for psychiatrists who may be working with Juvenile HD, and Gibson et al. [23] give guidance on these symptoms to psychiatric nursing colleagues working in the community.

### Psychology and psychotherapy

Affected persons with HD experience a change in mental state as a response to the illness and life events, just like populations without neurodegenerative disease. To reflect this, Simpson et al. [24] have produced guidance drawn from Anderson’s (2018) guidelines [21] and hosted by the British Psychological Society (BPS), which can be delivered across professions. Importantly these also recognise the needs of HD carers; due to the paucity of research which recognises the impact of neuropsychiatric symptoms and cognition on engagement, Zarotti et al. [25] call for the development of equal access to more psychologically oriented interventions.

### Neuropsychology and cognitive rehabilitation

Cognitive deficits are detectable up to 10 years before motor symptoms onset [5]. Early assessment of cognitive changes, including alterations in psychomotor speed, executive skills, memory, emotion processing, and social cognition [26], are vital, particularly when these may be causing conflict in daily occupation [27]. Despite cognitive deficits being recognised as one of the main clinical manifestations, no formal guidelines or recommendations are available for neuropsychological assessment [28] and treatment of these symptoms [29]. However, multidisciplinary programs, including cognitive rehabilitation, have been shown to improve cognition in pre-manifest and manifest HD patients [30, 31]. Moreover, a randomised study of computerised cognitive training has been recently carried out, providing feasibility and acceptability of this kind of intervention and promoting additional studies in this area [32].

### General practitioner

The challenges in the structure of health and social care in which they live may not always allow the person with HD and their families access to specialist support. The General Practitioner (GP) might be the only medical practitioner they encounter. Neither party may feel they can offer anything of value to such a rare and complex disorder. The review by El Nimr & Barrett, although not a guideline, outlines that GPs can provide valuable support to patients and their families [33]. It is relevant to all areas as it highlights key considerations that can be overlooked if one is not familiar with the disease. A more detailed physician's guide is freely accessible on the website of the Huntington's Disease Society of America (HDSA) [34].

### Genetic counselling

Soon after identifying the *HTT* gene, a simple and accurate blood test became available in clinical practice, so the first guideline for genetic testing and counselling was established [35]. The requirements for genetic counselling differ for diagnostic and presymptomatic (predictive) testing. Predictive testing is governed by precise guidance on the content of counselling and the performance of genetic testing [36]. The main emphasis is to ensure that people at risk of inheriting HD get support and guidance to make the best-informed decision on whether to undergo the genetic test or not. Genetic counselling should aim to prepare the at-risk person and their companions to handle the test outcome and plan for follow-up support after the test process is done. The variety and complexity of clinical situations in which diagnostic genetic testing for HD may be required and differential diagnosis make it impractical to establish guidelines such as those for predictive testing [37].

### Nursing

Nurses are acknowledged as an important part of delivering care for people with HD and their families [38]. They are highly valued for their knowledge, consistent presence, and case management [39]. So perhaps it is surprising that there are no standardised guidelines for nursing within the peer-reviewed and grey literature. However, as Baker et al. [40] note, nursing goes beyond case management and clinic coordination. They advocate strongly for the role to become recognised as it has been in other neurodegenerative diseases through defining the role. Although nursing has been shown to be important at all stages of the disease, it is more evident in the middle and later stages of the disease, when nursing and skilled nursing care become the predominant need.

From the national evidence of many European countries (e.g., Germany and the Netherlands), we know that special nursing knowledge is often based on many years of experience in the care of HD patients and that this nursing expertise is concentrated in the facilities that are intensively involved in the care of HD. In order to further expand the important role of nursing as a profession and its care services and to bring them more into a scientific focus, the targeted use of specially trained nurses is recommended, among others, according to the Advanced Practice Nursing (APN) model. According to Hamric & Tracy [41], these are academically trained nursing experts at the master's level. They have a specialised, extended competence profile and work with a focus on the individual care needs of affected families in all sectors of the healthcare system. They use current knowledge and develop it further independently. The APN works in outpatient and inpatient settings as part of the MDT and forms the nursing interface between these fields.

### Case management

Case management is the coordination and meeting of individual and disease-specific needs. The greatest evidence for the importance of case management in the literature we referred to earlier was concerning our nursing colleagues. Still, in line with Simpson and Rae [1], the key to the value of case management is not profession-specific but consistent presence with knowledge of the disease, the individual and the system of the individual.

### Social work

In discussions in our networks, it was felt that intercountry differences were at their greatest in this area. Whilst there are no guidelines, a seminal paper by Yale and Martindale [42] indicates that social workers are key personnel when, particularly in the navigation and provision of community social support, as do Simpson and Rae [1].

### Physiotherapy

Physiotherapy interventions are a mainstay of multidisciplinary care and have been demonstrated to be safe and beneficial for people living with HD. As part of a recent guideline document, Quinn et al. [43] noted strong evidence for aerobic exercise, alone or in combination with resistance training, for maintaining cardiorespiratory fitness and motor function. Strong evidence also existed for supervised gait training to address deficits in stride length and gait speed. Weak evidence was found for respiratory muscle training to maintain pulmonary function. There was also weak evidence supporting a positive effect of exercise training on balance but not falls. Evidence stemmed from clinical trials in people living with presymptomatic to moderate HD.

### Occupational therapy

An Occupational Therapist (OT) will seek to address the multiple facets of HD which strengthen or hinder the person’s performance in any of their activities of daily living (ADL) throughout their life span at a personal, environmental, and occupational level. As with many of the professions crucial to the success of MDT working in HD care, the scientific evidence documented for the outcomes of OT is sparse, but the value and scope of OT are acknowledged [44] with clear illustrations of the importance of assessing the functional manifestation of HD by Cook et al. [45] and the consequences of not. Best practice guidelines were produced in 2012 to establish a standard of care, and from this summary, guidance is in the form of clinical tips (hda.org.uk). There are ongoing plans to update the clinical tips and standard of guidelines and stimulate scientific evidence.

### Dietician and nutritional support

Critical to the overall well-being of the person with HD at any stage of their condition is their nutrition and calorific intake. Brotherton et al.’s [46] Nutritional Standards Guidelines still represent the essence of specialist nutritional practice, whether utilised through the philosophy of dietetics or nursing. They also sit alongside the work undertaken jointly and internationally with Speech and Language Therapists on the ISSDI framework for managing dysphagia which unifies the language of nutritional texture. Brotherton et al. again point to the need for MDT discussion, particularly in advancing diseases when the cognitive status affects how nutrition is delivered.

### Speech and language therapy

The two guidelines on managing swallowing, speech, language, and communication difficulties emphasise the importance of early Speech and Language Therapy (SLT) referral as good practice [47, 48]. Abnormalities in all phases of swallowing and throughout the disease progression have been identified, and the use of instrumental assessment in combination with the clinical evaluation should be performed regularly for appropriate intervention and to ensure the safety and efficiency of oral feeding. Furthermore, the SLT contributes to the multidisciplinary discussion about non-oral feeding options. Motor speech and language difficulties are detectable since the early stage of HD, resulting in severe communication impairment in later stages. A comprehensive assessment of these difficulties is recommended throughout the disease to evaluate the communication needs of the HD patient and family. Early implementation of communication strategies, including augmentative and alternative communication, and other interventions should be considered to improve the quality of life and patient participation.

### Oral healthcare and dentistry

Due to time constraints and specialist training (for dentistry for those that deliver care), the oral care of people with HD may be neglected. As Rae et al. [49] point out, this creates an uncomfortable reality for patients, carers, families, and professionals. Furthermore, in an update on the literature on the oral manifestations of HD, Munhoz et al. [50] point to the systemic ramifications of a lack of oversight on the overall health of the individual but also oral health in HD as a marker which may determine other aspects of the illness. Manley et al.’s [51] Oral Care Guidelines seek to make this crucial need a proactive rather than a reactive measure, demonstrating the need for discussion between dentistry, dietetics and nutritional care professionals.

### Other professions

Additional to the MDT’s core professions, others such as the pastoral caretaker or art and music therapist are just as essential. No specific guidelines are available for these professions, but proof of concept studies about HD hold promise [52].

Sleep specialists exist in some services as disturbances are a common and debilitating aspect of HD that adversely impact activities of daily living and quality of life [53]—still, multiple members of the MDT who address it, such as neuropsychiatrists or occupational therapists. Despite the negative consequences of sleep disturbances, the application of sleep science in HD is only beginning to gain traction. However, for now, Anderson’s neuropsychiatry guidelines [21] recommend a holistic, comprehensive assessment care approach that looks at the person with HD.

## Discussion and positioning

In this position paper, we have shown that the complexity of the disease, the lay associations, the voice of the patients and the global regulations endorse the inter- and multidisciplinary approach to the treatment and care of HD patients and families. Thus, there is no longer a need to prove the "state of the art". In our position statement, we illustrate the available guidelines and point out guidelines still missing in this field. In some areas of HD, the available guidelines are based on expert consensus. Killoran argues that good clinical care, as described by expert consensus, should not be put on hold due to the lack of population-based evidence approaches to a disease as multifaceted and complex as HD, as this could lead to real harm at the individual level (54). Currently, the guidelines or further plans to update them are not organised by a central body and a coordinating entity to make them available to all who need information and guidance on multidisciplinary care in HD. This position paper reveals that there are key essential professional groups involved in the MDT who do not have an HD guideline available, like psychologists, social workers, nurses, case managers and general practitioners. Most professionals working with HD are subjected to commissioning disparity but also research inequity. In contrast to the exciting research opportunities in the field of disease curation, it is difficult to find funding opportunities for the topics of care, nursing and management of the day-to-day clinical problems of HD patients and families. Nevertheless, all the authors of this paper are performing research, each within their own discipline, adding evidence to the guidelines. Therefore, we propose to update guidelines every 3–5 years and to share this update from a central point in the global scientific world. It is of key importance to share evidence-based approaches based on expert consensus with the field and make visible the evidence to local health and social care commissioners wherever that care is taking place.

In addition to this call for more research and communication on available knowledge and practice, there is a special disease-specific feature for collaborating within an HD MDT. As the patient frequently does not recognise symptoms the way the family experiences them, the patient is inclined to repel several kinds of treatment and care. The rebuttal of the patient might be fierce and mistaken for autonomy leading to the withdrawal of professional involvement. The undesired side effect might be that the family is left alone with their needs and seeking appropriate help and guidance. Moreover, the patient is left alone with a neurodegenerative brain disorder coinciding with dwindling reflective capacities. In these complex circumstances, a multidisciplinary team should rely on several qualities –flexibility in one’s own professional role, perseverance in aiming for a bond of trust despite rejection and stepping in for one another. Sometimes a speech and language therapist may reach a bond of trust with the patient and is, therefore, able to encourage a patient to take the medication prescribed by the psychiatrist and improve compliance with the required treatment. A few weeks later, it might occur that the social worker has a proper entrance to the system, leading to adherence to a small part of the treatment plan. As a member of a multidisciplinary team, it is pivotal to know the whole treatment and care plan and advocate for it. Whilst stepping in for a colleague is sometimes difficult, it might be the best available way to help the patient and family. This approach must be shared by the whole team to ensure success, and interdisciplinary support within the team is crucial. Flexibility and a sense of humour facilitate teamwork. A prerequisite for a multidisciplinary team is a shared view on treatment and care for HD patients and their families. Expert knowledge of HD and the ability to acknowledge and deal with divergent experiences within a system approach are components of successful treatment and care for HD families.

The objective of this paper is to draw attention to the health needs of HD families in general and promote the use of appropriate specific interventions for disease therapy and management. It also evaluates and endorses the value of the existing guidelines.

## Conclusions

As Huntington's disease presents a high complexity of symptoms, ranging from behavioural disorders to a variety of motor complaints to cognitive deficits, occurring in varying severity and changing during the course of the disease, multidisciplinary care and treatment are absolutely essential to meet the individual needs of patients and their families. The multidisciplinary team should be composed of different professionals who work closely together and interact within the group. It is also important to establish an individualised care and treatment plan for every single case of a patient and family. A case manager or coordinator may facilitate the work of the multidisciplinary team. There are already several guidelines and expert recommendations for different professional groups. However, for many professional groups, there is a lack of evidence to draw up the guidelines. Time needs to be invested in developing missing guidelines. Although it is important to gain further insights and evidence, we should start to use and implement existing knowledge and expertise in clinical practice and promote interdisciplinary care and treatment of HD patients and their families in the best possible way, regardless of where a patient's care takes place.

## Data Availability

Not applicable.

## Abbreviations

ADL: Activities of daily living
APN: Advance practise nursing
BPS: British Psychological Society
EBM: Evidence-based medicine
EHA: European Huntington Association
EHDN: European Huntington’s Disease Network
ERN-RND: European Reference Network for Rare Neurological Diseases
GP: General practitioner
HCP: Health care provider
HD: Huntington’s disease
HDSA: Huntington's Disease Society of America
MTC WG: Multidisciplinary Treatment and Care Working Group
MDT: Multidisciplinary Team
OCD: Obsessive compulsive behaviour
OT: Occupational therapist
SLT: Speech and language therapy
WHO: World Health Organisation
